# Surgery-related disseminated intravascular coagulation predicts postoperative complications

**DOI:** 10.1186/s12893-023-01986-9

**Published:** 2023-04-11

**Authors:** Yuki Imaoka, Masahiro Ohira, Kouki Imaoka, Tomoaki Bekki, Ryosuke Nakano, Shintaro Kuroda, Hiroyuki Tahara, Kentaro Ide, Tsuyoshi Kobayashi, Yuka Tanaka, Hideki Ohdan

**Affiliations:** 1 Department of Gastroenterological and Transplant Surgery, School of Biomedical and Health Sciences Hiroshima University, Hiroshima University, 1-2-3 Kasumi, Minami-Ku, Hiroshima, 734-8551 Japan; 2 Division of Regeneration and Medicine,, Medical Center for Translational and Clinical Research, Hiroshima University Hospital, 1-2-3 Kasumi, Minami-Ku, Hiroshima, 734-8551 Japan

**Keywords:** Disseminated intravascular coagulation, Surgery, Postoperative complication

## Abstract

**Purpose:**

The rate of postoperative morbidity, including infectious complications, is still high after major hepatobiliary pancreatic (HBP) surgery. Although surgery-related disseminated intravascular coagulation (DIC) occurs in some cases, its significance has not been elucidated in HBP surgery. This study aimed to evaluate the influence of surgery-related DIC on the complication severity after HBP surgery.

**Methods:**

We analyzed the records of 100 patients with hepatectomy in two or more segments, hepatectomy with biliary tract reconstruction, and pancreaticoduodenectomy. The baseline characteristics and complications were compared between patients with and without surgery-related DIC on postoperative day 1 (POD1) after HBP surgery between 2010 and 2018. Complication severity was assessed using the Comprehensive Complication Index (CCI).

**Results:**

The DIC group (surgery-related DIC on POD1) had predictive factors, such as larger bleeding volume and higher liver enzyme levels. The DIC group exhibited significantly elevated rates of surgical site infection, sepsis, prolonged intensive care unit stay, more frequent blood transfusions, and higher CCI. Furthermore, compared with and without adjustment of DIC, odds ratio (OR) of AST level and operation time for  the risk of high CCI decreased (OR of AST level: 1.25 to 1.19 and OR of operation time: 1.30 to 1.23) and the significant differences had vanished.

**Conclusions:**

Surgery-related DIC on POD1 could be a partial mediator between AST level, operation time and higher CCI. The prevention or proper management of surgery-related DIC on POD1 can be an important target to reduce the severity of postoperative complications.

## Introduction

Major hepatobiliary pancreatic (HBP) surgery is known to be associated with a high risk of postoperative complications. Although the mortality rate is low, the morbidity rates still range from 40–75% [1–5]. Hence, surgeons should anticipate high rates of morbidity and control the associated risks to prevent complications.

Disseminated intravascular coagulation (DIC) occurs in various clinical conditions, including sepsis, trauma, cancer, and immunological disorders [6]. Guidelines for the diagnosis and treatment of DIC were recently published in Britain [6], Japan [7], and Italy [8]. Although surgery-related DIC occasionally occurs after invasive surgery, including hepatobiliary and pancreatic surgery [9], the significance of surgery-related DIC has not yet been determined.

The objective of this study was to evaluate the influence of surgery-related DIC on the severity of complications after major HBP surgery.

## Methods

### Patients

We performed 149 major HBP surgeries in our unit between January 2010 and October 2018, excluding cases that involved liver transplantation and liver donation. We analyzed 100 major HBP surgeries that we could identify as involving surgery-related DIC. Data concerning patient characteristics at the time of surgery (age, gender, primary disease, surgical procedure, body mass index, diabetes, preoperative biliary drainage, cholangitis in medical history), surgical factors (operation time, bleeding volume), levels of total bilirubin (T-bil), aspartate transaminase (AST), alkaline phosphatase (ALP), alkaline transferase (ALT), albumin, Albumin-Bilirubin (ALBI) score [10], international normalized ratio of prothrombin time (PT-INR), white blood cell (WBC), hemoglobin, and platelet, prior to surgery, and short-outcomes were collected from electronic records. This study has been reported in line with the STROCSS criteria [11].

### Preoperative biliary drainage

Preoperative biliary decompression was performed to reduce serum bilirubin concentrations for all patients with jaundice and to control segmental cholangitis. A biliary stent, percutaneous transhepatic biliary drainage, or endoscopic nasobiliary drainage was used for drainage. Prophylactic antibiotics were administered for three days postoperatively according to sensitivity results of a preoperative biliary culture test.

### HBP surgery

Major HBP surgery was defined to contain pancreaticoduodenectomy (PD), hemihepatectomy or greater, and hepatopancreaticoduodenectomy [12].

### The DIC score

The DIC score established by the Japanese Association for Acute Medicine (JAAM DIC diagnostic criteria) was also assessed on postoperative day 1 (POD1). The scoring system used to diagnose DIC was developed by JAAM in 2006 [13]. The revised scoring system for DIC that is used in Japan is shown in Table 1. Surgery-related DIC on POD1 was diagnosed when (1) the DIC score was 4 points or more on POD1 and (2) the DIC score was 3 points or less before surgery. All patients were followed up for 30 days after enrollment into the study, and the incidence rate of the preoperative complications at 30 days was assessed. All complications were evaluated using the Clavien-Dindo classification [14]. The disease was classified as grade II or higher if complications were present. The Comprehensive Complication Index (CCI) was finally calculated as the sum of all complications that were weighted for their severity by patients and physicians, with the final formula yielding a score that ranged from 0 (no complication) to 100 (death) [15]. Severe morbidity was defined as CCI ≥ 40, as described in a previous report [16].

**Table 1:** DIC diagnostic criteria defined by the Japanese Association for Acute Medicine

Characteristics	Score
Systemic inflammatory response syndrome criteria	
≥ 3	1
0–2	0
Platelet count, × 10^9^/L	
< 80 or > 50% decrease within 24 h	3
≥ 80 and < 120, or > 30% decrease within 24 h	1
≥ 12	0
Prothrombin time (value of patient/normal value)	
≥ 1.2	1
< 1.2	0
Fibrin/fibrinogen degradation products, mg/L	
≥ 25	3
≥ 10 and < 25	1
< 1	0
Diagnosis ≥ 4 points	DIC

*DIC* disseminated intravascular coagulation

The CCI can easily be calculated online, with free access at www.assessurgery.com. Follow-up results were obtained from medical records and the primary physician interviews.

### Statistical analysis

JMP statistical software (JMP® 14; SAS Institute Inc., Cary, NC, USA) was used for all statistical analyses. Data were summarized using median values and interquartile ranges (IQR) for continuous variables, and number and percentage values for categorical variables. The Mann–Whitney U test was used in analyzing all continuous variables and Pearson’s Chi-squared test was used to determine the significance of differences between categorical values. Fisher’s exact test was used when a table included a cell with an expected frequency of < 5. The incidence curves of patients with a high CCI (≥ 40) were compared using the logistic analyses as a univariate analysis. The well-known factors such as operation time, bleeding time and patient age as a risk for the occurrence of postoperative complications were selected in a multivariate analysis. Bleeding volume and operative time were strongly correlated, therefor bleeding volume was excluded. Regarding a liver enzyme such as AST level, AST values were included in the analysis continuous variables due to the strongly correlation with AST values and postoperative complications in univariate analysis. DIC on POD1 was analyzed as a mediator to connect exposures such as surgical invasiveness and preoperative patients’ condition with high CCI, using the causal mediation analysis [17]. To evaluate the influence of surgery-related DIC as a mediate factor of high CCI, multivariate analyses of risk of high CCI with and without adjustment of DIC were performed. A *p*-value of less than 0.05 was considered statistically significant in all analyses.

## Results

### Correlation between surgery-related DIC and clinical characteristics

We analyzed the records of 77 men and 23 women with an overall median age at operation of 69.5 years [64–77]. The distribution of patients’ characteristics is shown in Table 2. The underlying diagnosis was hepatocellular carcinoma (HCC) in 42 cases (42%), pancreatic cancer in 8 cases (8%), duodenal cancer in 14 cases (14%), and cholangiocarcinoma in 22 cases (22%). We first divided the patients into two groups according to the presence of surgery-related DIC on POD1 (Table 3). Patients with surgery-related DIC on POD1 (DIC group) had a significantly increased bleeding volume, and higher AST and ALT levels before surgery. However, there were no significant differences in preoperative conditions, including prior cholangitis and biliary drainage.

**Table 2:** Patient characteristics

Variables	*n* = 100
Age (years)	69.5 [64–77]
Male/Female	77/23
Diagnosis	
HCC	42 (42%)
Pancreatic cancer	8 (8%)
Cholangiocarcinoma	22 (22%)
Duodenal cancer	14 (14%)
Gall bladder cancer	5 (5%)
Combined hepatocellular and cholangiocarcinoma	2 (2%)
Biliary injury	1 (1%)
Duodenal gastrinoma	1 (1%)
IPMN of pancreas	1 (1%)
Others	4 (4%)
Surgical procedure	
+ Pancreaticoduodenectomy	27 (27%)
+ Liver resection	74 (74%)
+ Biliary tract reconstruction	47 (47%)

*HCC* hepatocellular cancer, *IPMN* intraductal papillary mucinous neoplasm

**Table 3:** Background data according to surgery-related DIC

	DIC	Non-DIC	
(%) or [IQR]	*N* = 24	*N* = 76	*P*-value
Male/ Female	22 (91.6)	55 (72.3)	0.05
Age (years)	69.5 [64–78]	69.5 [64–77]	0.79
BMI (kg/m^2^)	21.9 [19.4–23.1]	21.9 [20.0–23.8]	0.68
Diabetes, yes	7 (29.1)	24 (31.6)	0.82
Preoperative biliary drainage	3 (12.5)	21 (27.6)	0.13
Cholangitis in medical history	1 (4.1)	4 (5.2)	1.00
Benign	0 (0)	5 (6.6)	0.33
Hepatectomy	19 (79.1)	55 (72.4)	0.51
Pancreatectomy	5 (20.8)	23 (30.2)	0.37
Biliary tract reconstruction	9 (37.5)	38 (50.0)	0.28
Lymph node dissection	11 (45.8)	41 (53.9)	0.49
Operation time (min)	470 [376–705]	441 [367–516]	0.27
Bleeding volume (mL)	936 [631–2,191]	547 [360–890]	< 0.01
T-bil (mg/dL)	0.6 [0.6–1.1]	0.6 [0.6–1.1]	0.83
AST(IU/L)	48 [31–74]	27 [21–43]	< 0.01
ALP (IU/L)	392 [285–595]	299 [227–463]	0.19
Albumin (mg/dL)	4.1 [3.8–4.5]	3.9 [3.5–4.5]	0.23
PT-INR (%)	1.1 [1.0–1.2]	1.1 [1.0–1.2]	0.23
WBC (/μL)	5,910[5,200–6,190]	5,860 [4,740–6,780]	0.75
Hemoglobin (mg/dL)	12.3 [11.0–14.3]	13.2 [12–14.6]	0.17
Platelet (× 10^4^/μL)	18.1 [13.3–26.6]	19.3 [15.6–24.1]	0.65
ALBI score	-0.42 [-0.50–0.32]	-0.43 [-0.50–0.32]	0.96

*DIC* disseminated intravascular coagulation, *BMI* body mass index, *T-bil* total bilirubin, *AST* aspartate transaminase, *ALT* alkaline transferase, *ALP* alkaline phosphatase, *CRP* C-reactive protein, *PT-INR* prothrombin time-international normalized ratio, *WBC* white blood cell, *ALBI score* Albumin Bilirubin sore

### Clinical outcomes based on surgery-related DIC

**Fig. 1:**
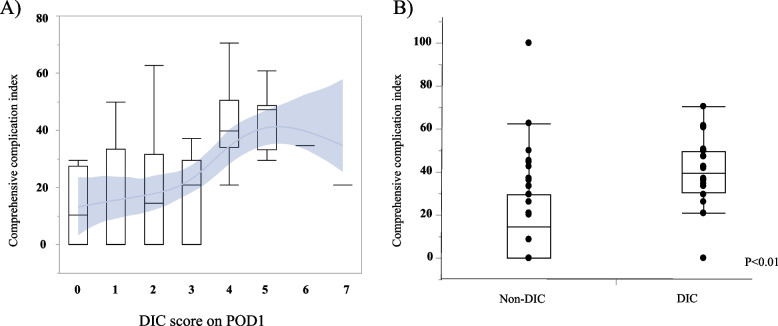
Comprehensive Complication Index due to DIC score. **A** Surgery-related DIC. **B** The presence of surgery-related DIC. DIC: disseminated intravascular coagulation

Table 4 shows the short-term outcomes based on the conditions of surgery-related DIC on POD1. Patients in the DIC group had significantly higher rates of surgical site infection (SSI), bloodstream infection, stays in an intensive care unit (ICU), red blood cells (RBC) infusion, fresh frozen plasma (FFP) infusion and 30-day morbidity compared with patients in the non-DIC group. Next, we analyzed the influence of surgery-related DIC on POD1 on postoperative complications using the CCI [15]. Patients with higher DIC score tended to increase the CCI, especially among patients more over 4 points of DIC score. (Fig. 1A) Patients with surgery-related DIC on POD1 had a significantly higher CCI than patients who did not develop surgery-related DIC on POD1 (38.5 vs 14.5, *p* < 0.01). (Fig. 1B).

**Table 4:** Relationship between surgery-related DIC and short-term outcomes

(%) or [IQR]	DIC 24 cases	Non-DIC 76 cases	*P*-value
SSI	11 (45.8)	16 (21.1)	0.02
Cholangitis	6 (25.0)	8 (10.5)	0.07
Bile leakage	2 (8.3)	9 (11.8)	1.00
Pancreatic Fistula	(16.7)	(5.3)	0.09
Bloodstream infection	5 (20.8)	4 (5.3)	0.03
ICU readmission rate	2 (8.3)	2 (2.6)	0.24
Postoperative bleeding	1 (4.2)	2 (2.6)	0.57
RBC infusion	7 (29.2)	9 (11.8)	0.04
FFP infusion	10 (41.7)	13 (17.1)	0.01
Stay in ICU (days)	2 [0–3]	1 [0–2]	< 0.01
Hospitalization periods (days)	22 [17–41]	19 [12-31]	0.19
30-day morbidity	23 (95.8)	41 (54.0)	< 0.01
90-day mortality	0 (0)	2 (2.6)	1.00

*DIC* disseminated intravascular coagulation, *SSI* surgical site infection, *ICU* intensive care unit, *RBC* Red blood cell, *FFP* Fresh frozen plasma

### Risk factors for surgery-related DIC on day 1

We analyzed risk factors for DIC on day 1. Univariable analysis showed that higher AST ^+10U/L^ level (OR; 1.24, 95% confidence interval; 1.06–1.45, *p* < 0.01), operation time^+1 h^ (OR; 1.20, 95% confidence interval; 1.02–1.42, *p* = 0.02), and bleeding volume^+100 ml^ (OR; 1.05, 95% confidence interval; 1.01–1.09, *p* = 0.01) had a relation with the risk of surgery-related DIC on day 1. Multivariate analysis revealed that the independent risk factors of surgery-related DIC were higher AST ^+10^ ^U/L^ level (OR; 1.23, 95% confidence interval; 1.05–1.45, *p* < 0.01), and operation time^+1^^h^ (OR; 1.22, 95% confidence interval; 1.02–1.46, *p* = 0.02), (Table 4).

### Risk factors for high CCI without and with adjustment of surgery-related DIC on day 1

We analyzed risk factors for severe morbidity via univariate and multivariate analysis. Univariate analysis revealed that surgery-related DIC on POD1 had strong relation for severe morbidities (OR; 9.86, 95% confidence interval; 3.23–30.07, *p* < 0.01). Univariate analysis even after adjustment with age revealed that surgery-related DIC on POD1 had strong relation for severe morbidities (OR; 9.81, 95% confidence interval; 3.20–30.09, *p* < 0.01). Multivariate analysis without adjustment of surgery-related DIC on day 1 revealed that higher AST ^+10 U/L^ level (OR; 1.25, 95% confidence interval; 1.06–1.47, *p* < 0.01), and operation time^+1 h^ (OR; 1.30, 95% confidence interval; 1.07–1.59, *p* < 0.01) had a strong relation with the risk of high CCI (Table 6). Multivariate analysis with adjustment of surgery-related DIC on day 1 also revealed that higher AST ^+10 U/L^ level (OR; 1.19, 95% confidence interval; 0.99–1.43, *p* = 0.06), operation time^+1 h^ (OR; 1.23, 95% confidence interval; 0.99.-1.53, *p* = 0.06), DIC on POD1 (OR; 6.12, 95% confidence interval; 1.82–20.47, *p* < 0.01) had a relation with the risk of high CCI (Table 6).

## Discussion

This study enrolled 100 major HBP surgeries to evaluate the impact of surgery-related DIC on the severity of complications using the causal mediation analysis. These results indicated that DIC could be detected as a partial mediator between higher AST level, prolonged operation time and higher CCI. Surgery-related DIC on POD1 was also significantly associated with surgical site infection, bloodstream infection, longer stays in an ICU, high CCI, and morbidity within 30 days. Our report will help to spread the awareness of surgery-related DIC and stimulate discussion of therapeutic interventions for surgery-related DIC such as the anticoagulant therapy.

The risk factors for surgery-related DIC were higher preoperative liver enzyme levels (OR; 1.23, 95% confidence interval; 1.05–1.45, *p* < 0.01), and longer operation time (OR; 1.22, 95% confidence interval; 1.02–1.46, *p* = 0.02). (Table. 4) This result showed that surgery-related DIC was strongly related to preoperative liver function and invasive surgery. Several indexes combined with preoperative liver enzymes such as the AST-to-platelet ratio index and AST-to-neutrophil ratio index have been shown to be useful as predictors of postoperative outcomes after hepatectomy [18–20]. Invasive surgery, such as intraoperative and postoperative bleeding and longer operation time was also well-established risk factors for high morbidity and poor survival among patients with colorectal liver metastases and hepatocellular carcinoma [21–25]. ALBI score was well-known as a predictive factor of posthepatectomy liver failure and poor outcomes [26, 27]. In our study, ALBI score had no relation with surgery-related DIC and high CCI. These reports supported that our finding that surgery-related DIC was strongly related to preoperative liver damage and invasive surgery.

Our findings also showed that surgery-related DIC on POD1 reflects the degree of invasion in patients that undergo major HBP surgery. We hypothesized that invasive procedures could cause clotting abnormality and that postoperative complications could be increased by delaying blood supply to organs after major HBP surgery. DIC is recognized as a severe condition involving the widespread activation of coagulation, which leads to blood clots and can decrease blood supply to organs [28]. The cardiovascular surgery-associated DIC was discussed in previous report [29]. However, surgery-related DIC is not still prevalent in the field of gastrointestinal surgery [30], and it is challenging to treat surgery-related DIC in Japan because of the risk of postoperative bleeding. However, only a few studies have reported that recombinant soluble thrombomodulin improved surgery-related DIC [30, 31]. Hashimoto et al. reviewed that anticoagulant therapy may be effective and safe in DIC after gastrointestinal surgery [32, 33]. The DIC score ≥ 5 postoperatively was reported as an independent risk factor for post-hepatectomy liver failure [34]. Surgery-related DIC may result in systemic inflammation and infection after invasive gastroenterological surgery. In addition, blood coagulation and fibrinolysis result in systemic thrombin and plasmin activation in vessels in DIC patients [35]. Therefore, we focused on the relationship between surgery-related DIC on POD1 and postoperative complications. It needs to be further discussed that a high DIC score on POD1 should have included decrease in PT and platelet number levels due to hepatectomy and does not reflect DIC status after operation completely. However, Table 4 showed that the DIC group had a high rate of using perioperative FFP and RBC infusions to improve coagulopathy, suggesting that surgeons carefully monitored coagulopathy postoperatively in the clinical setting. To evaluate the influence of surgery-related DIC, which is as a mediate factor, on high CCI clearly, DIC on POD1 wea analyzed as a mediator to connect exposures such as surgical invasiveness and preoperative patients’ condition with high CCI, using the causal mediation analysis [17].Table .6 shows that DIC had a strong relation with high CCI. (OR:9.86) Higher AST level and operation time had a relation with surgery-related DIC and high CCI in Tables 5 and 6. Compared with and without adjustment of surgery-related DIC, OR of AST level and operation time decreased (OR of AST level: 1.25 to 1.19 and OR of operation time: 1.30 to 1.23) and the significant differences had disappeared. These results indicated that DIC could be a partial mediator between AST level, operation time and high CCI.

**Table 5:** Risk factors for DIC on day 1 according to univariate and multivariate analyses

	Univariate analysis			Multivariate analysis		
Subject	OR	CI	*P*-value	OR	CI	*P*-value
Gender Male	4.20	0.91–19.44	0.07			
Age, years	1.01	0.96–1.06	0.66	1.02	0.96–1.08	0.57
BMI, kg/m^2^	0.96	0.83–1.11	0.58			
Diabetes	0.89	0.32–2.43	0.82			
Preoperative biliary drainage	0.37	0.10–1.38	0.14			
Cholangitis in medical history	0.78	0.08–7.36	0.83			
Preoperative blood test						
ALBI score	0.92	0.04–1.08	0.96			
AST^+10^, U/L	1.24	1.06–1.45	< 0.01	1.23	1.05–1.45	< 0.01
PT,	6.72	0.19–238.3	0.30			
Platelet. 10^4^/μl	1.00	0.94–1.07-	0.91			
Operation factor						
Biliary tract reconstruction	0.60	0.23–1.53	0.29			
Lymph node dissection	0.72	0.28–1.81	0.48			
Operation time, h	1.20	1.02–1.42	0.02	1.22	1.02–1.46	0.02
Bleeding volume ^+100^, ml	1.05	1.01–1.09	0.01			
Hepatectomy	1.45	0.48–4.39	0.50			

*DIC* disseminated intravascular coagulation, *BMI* body mass index, *AST* aspartate transaminase, *PT-INR* international normalized ratio of prothrombin time

**Table 6:** Risk factors for high CCI according to univariate and multivariate analyses

	Univariate analysis			Multivariate analysis without adjustment for DIC			Multivariate analysis with adjustment for DIC		
Subject	OR	CI	*P*-value	OR	CI	*P*-value	OR	CI	*P*-value
Preoperative factor									
Gender Male	2.98	0.63–13.98	0.16						
Age, years	1.02	0.96–1.09	0.60	1.04	0.97–1.11	0.27	1.04	0.96–1.12	0.34
BMI, kg/m^2^	0.93	0.80–1.10	0.41						
Diabetes	1.03	0.33–2.84	0.95						
Biliary drainage	2.20	0.75–6.43	0.15						
Cholangitis in medical history	3.05	0.47–19.74	0.24						
ALBI score	9.42	0.42–210.92	0.16						
AST^+10^, U/L	1.26	1.08–1.47	< 0.01	1.25	1.06–1.47	< 0.01	1.19	0.99–1.43	0.06
Operation factor									
Biliary tract reconstruction	2.25	0.80–6.31	0.12						
Lymph node dissection	2.33	0.81–6.74	0.12						
Operation time, h	1.26	1.05–1.50	< 0.01	1.30	1.07–1.59	< 0.01	1.23	0.99–1.53	0.06
Bleeding volume ^+100^, ml	1.02	0.98–1.06	0.24						
Hepatectomy	0.98	0.31–3.05	0.97						
Perioperative factor									
DIC on day 1	9.86	3.23–30.07	< 0.01	-	-	-	6.12	1.82–20.47	< 0.01

*DIC* disseminated intravascular coagulation, *BMI* body mass index, *AST* aspartate transaminase

In 2013, the CCI was developed and has been reported as an accurate index that reflects the severity of all postoperative complications [15]. The CCI summarizes the postoperative experience with respect to the development of complications using the Clavien-Dindo classification system [36]. Nakanishi et al. reported that massive intraoperative blood loss and combined PD were independent risk factors for CCI ≧ 40 among patients that underwent major hepatectomy for biliary cancer [16]. In this study, surgery-related DIC on POD1 led to an increase in several severe complications and an elevated CCI. Generally, DIC should be treated promptly to resolve the pathological conditions associated with DIC [37, 38]. Optimized surgical decisions are needed to minimize the risk of massive blood loss and a longer operation time [16]. Additionally, a reduction in surgical invasiveness and treatment of surgery-related DIC could prevent development of high CCI conditions in patients after major HBP surgery.

Although this study presents the risk factors for severe complications among major HBP surgery, it is limited by its small sample size, short period of observation, and single-center design. In addition, many unknown issues involving surgery-related DIC on POD1, including the relationships between DIC and complications, remain undetermined. Larger cohorts, including multi-institutional joint research, are necessary to investigate the relationship between surgery-related DIC on POD1 and the severity of complications after major HBP surgery and add sub-analyses separated in hepatectomy and pancreatectomy. The results in this study can provide a good opportunity to focus on surgery-related DIC for many surgeons.

## Conclusions

Surgery-related DIC on POD1 could be a partial mediator between AST level, operation time and higher CCI. The prevention or proper management of surgery-related DIC on POD1 can be an important target to reduce the severity of postoperative complications.

## Data Availability

The datasets generated and/or analyzed during the current study are not publicly available due to institutional policies but are available from the corresponding author on reasonable request.

## Abbreviations

HBP: Major hepatobiliary pancreatic
DIC: Disseminated intravascular coagulation
T-bil: Total bilirubin
AST: Aspartate transaminase
ALBI score: Albumin-Bilirubin score
ALP: Alkaline phosphatase
ALT: Alkaline transferase
PT-INR: International normalized ratio of prothrombin time
WBC: White blood cell
PD: Pancreaticoduodenectomy
JAAM: The Japanese Association for Acute Medicine
CCI: Comprehensive Complication Index
HCC: Hepatocellular carcinoma
RBC: Red blood cells
FFP: Fresh frozen plasma
