# Correction: Surgery‑related disseminated intravascular coagulation predicts postoperative complications

**DOI:** 10.1186/s12893-023-02073-9

**Published:** 2023-06-24

**Authors:** Yuki Imaoka, Masahiro Ohira, Kouki Imaoka, Tomoaki Bekki, Ryosuke Nakano, Shintaro Kuroda, Hiroyuki Tahara, Kentaro Ide, Tsuyoshi Kobayashi, Yuka Tanaka, Hideki Ohdan

**Affiliations:** 1 Department of Gastroenterological and Transplant Surgery, School of Biomedical and Health Sciences, Hiroshima University, Hiroshima University, 1-2-3 Kasumi, Minami-Ku, Hiroshima, 734-8551 Japan; 2 Division of Regeneration and Medicine, Medical Center for Translational and Clinical Research, Hiroshima University Hospital, 1-2-3 Kasumi, Minami-Ku, Hiroshima, 734-8551 Japan

**Correction: BMC Surg 23, 86 (2023)**

**https://doi.org/10.1186/s12893-023-01986-9**

Following publication of the original article [1], In Fig. 1b, the label has been interchanged and the updated figure is shown below:

**Fig. 1:**
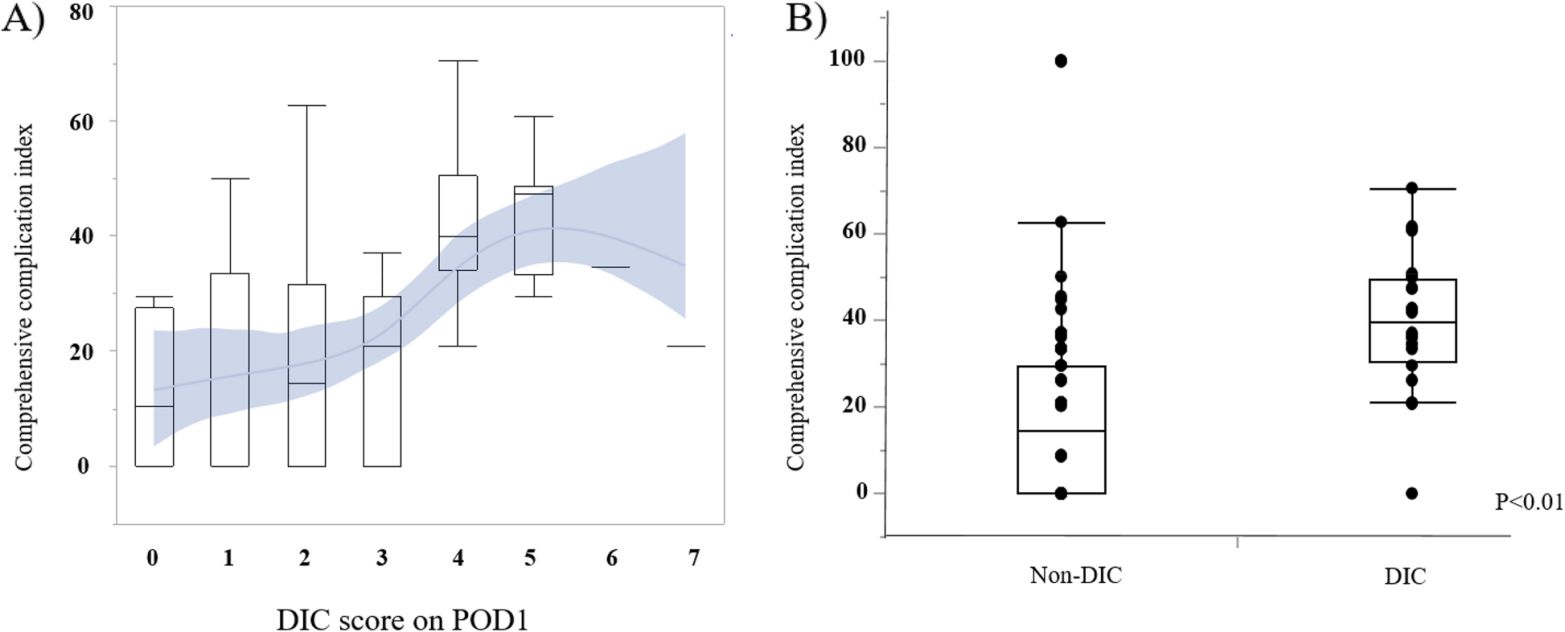
Comprehensive complication in dex due to DIC score

The original article has been corrected.
